# Research trends in biomimetic medical materials for tissue engineering: 3D bioprinting, surface modification, nano/micro-technology and clinical aspects in tissue engineering of cartilage and bone

**DOI:** 10.1186/s40824-016-0057-3

**Published:** 2016-05-04

**Authors:** Cen Chen, Sumi Bang, Younghak Cho, Sahnghoon Lee, Inseop Lee, ShengMin Zhang, Insup Noh

**Affiliations:** Bio-X Center, College of Life Sciences, Zhejiang Sci-Tech University, Hangzhou, People’s Republic of China; Seoul National University of Science and Technology, 232 Gongneung-roNowongu, Seoul, 11811 Republic of Korea; Department of Mechanical System Design Engineering, Seoul National University of Science and Technology, 232 Gongneung-ro, Nowon-gu, Seoul 11811 Republic of Korea; Department of Orthopaedic Surgery, Seoul National University College of Medicine/Seoul National University Hospital, Seoul, 110-799 Republic of Korea; Institute of Natural Sciences, Yonsei University, Seoul, 03722 Korea; Advanced Biomaterials and Tissue Engineering Center, Huazhong University of Science and Technology, Wuhan, P. R. China; Department of Chemical and Biomolecular Engineering, Seoul National University of Science and Technology, 232 Gongneung-ro, Nowon-gu, Seoul 01811 Republic of Korea

## Abstract

This review discusses about biomimetic medical materials for tissue engineering of bone and cartilage, after previous scientific commentary of the invitation-based, Korea-China joint symposium on biomimetic medical materials, which was held in Seoul, Korea, from October 22 to 26, 2015. The contents of this review were evolved from the presentations of that symposium. Four topics of biomimetic medical materials were discussed from different research groups here: 1) 3D bioprinting medical materials, 2) nano/micro-technology, 3) surface modification of biomaterials for their interactions with cells and 4) clinical aspects of biomaterials for cartilage focusing on cells, scaffolds and cytokines.

## Introduction

Scaffolds with bimimetics have been developed for tissue engineering, based on the sciences and engineering of biomaterials and nano/microstructures of defect tissues. The bone and cartilage tissues function according to the physicochemical properties and nano/micro-structures of tissues. Surface and bulk morphologies, defect sizes, extracellular matrix (ECM) domains, biological molecules and ECM structures are typical examples of major components and properties of defect tissues, which require tissue regeneration. 3D bioprinting among many technologies in tissue engineering has been applied to fabrications of scaffolds based on understanding of tissue morphologies and physicochemical properties in nano/micro levels, even though 3D bioprinting has at current stages many huddles to be overcome to its clinical applications. Tissue engineering of 3D printing scaffolds could be clinically accomplished by combining with (stem) cells, possibly with biological agents. In this review, we in different research groups have briefly discussed recent research trends in biomimetic medial materials for tissue engineering such as 3D bioprinting, surface modification, nano/micro-technology and clinical aspects in tissue engineering of bone and cartilage. Their backgrounds are designs of biomaterials in nanotechnology, fabrications of tissue engineering, surface modification and orthopedic clinics.

### Research trends of biomimetic medical materials: 3D bioprinting

Tissue engineering is repairing or regenerating diseased or damaged tissues and organs through implantation of the combinations of tissue engineering factors such as cells, scaffolds and biological signals with mechanical stimuli. Tissue engineering scaffolds provide primarily 3D structures of specific tissues and support tissue regeneration via cell adhesion, proliferation, and cellular communications and interactions. Porous polymer scaffolds for bone and cartilage regeneration have been traditionally fabricated through the methods of gas forming, soluble particle leaching and freeze drying [1–3]. Those methods have been developed by using functional chemistries and surface modifications of polymeric biomaterials. Porous scaffolds of biodegradable poly(lactide-*co*-glycolide) were fabricated by gas foaming of porogens such as ammonium bicarbonate particles. Injectable hydrogel was developed by using click chemistry, showing its advantages such as local delivery of bioactive molecules, easy handling, minimal invasiveness, and its potential applications to 3D bioprinting materials [4].

Fabrications of tissue engineering scaffolds have been recently focused on both utilization of biomimetic of extracellular matrix (ECM) and control of both cellular behaviors and biosignals. Novel fabrication methods of well-defined scaffolds have been reported for large tissue defects and organ replacement by using acellular scaffolds or cellular constructs [5, 6]. While acellular scaffolds were fabricated using various techniques such as heat, chemicals, light and molding, cellular constructs were done by combinations of cells and mostly photo-printing of hydrogels in 3D printing to mimic the properties of ECM of bone and cartilage. 3D printing of scaffolds was achieved by the methods of material extrusion, material jetting, binder jetting, sheet lamination, vat photo-polymerization, powder bed fusion and direct energy deposition [7, 8]. These methods have their own pros and cons in their applications to fabricating tissue engineering scaffolds. Stereolithography and fused deposition methods have disadvantages such as difficulty in bioprinting of precursor polymer solutions with live cells due to their damages during printing and side effects of cross-linking agents during hydrogel formation [9, 10]. In the case of vat photo-polymerization, active and non-toxic cross-linking agents are required for hydrogel fabrications, and at the same time, structural integrity of implanted scaffolds between scaffold biodegradation and tissue regeneration is important over in vivo implant periods. Syringe extrusion of cells complex during inkjet 3D printing is achieved by the methods of air pressure, syringe pump and screw, and cells-containing hydrogel is currently printed out with a dimension of 100 μm – 1 mm ranges in diameters [11, 12]. While those extrusion methods have advantages such as mechanical properties and possibility of combinations of biomaterials and cells, they also have limited properties such as shear stress to the wall of a nozzle tip and limited choices of hydrogel materials for cell encapsulation, leading to cells aggregation and damages.

Diverse biomaterials have been developed in 3D bioprinting of scaffolds [13–16]. While natural polymers such as collagen, hyaluronic acid, chitosan, cellulose, fibrin, alginate, agarose, gelatin, laminin and matrigel have been employed in 3D bioprinting through their chemical modifications, poly(ethylene glycol) poly(lactide-*co*-glycolide), polylactide, polycarprolactone, polyglycolide and poly(vinyl alcohol) have been employed as synthetic polymers for tissue engineering of bone and cartilage. Applications of biomimetics to hydrogel fabrication in bioprinting were possible by chemical modifications of biocompatible polymers such as poly(ethylene glycol), polycarbonates and poly(vinyl alcohol) among many synthetic medical polymers. Functional cross-linking of the polymers were possible through click chemistries such as photo- and thermal chemistry, Michael type addition reactions, pHs and others [17–21]. Inorganic biomaterials have been also employed for 3D printing by controlling the properties of 3D printers such as fabrication methods and their nozzle sizes and surface properties [22, 23]. Bioactive glass, hydroxyl apatite, biphasic calcium phosphate, hydroxyphosphate calcium and tricalcium phosphate were normally employed as inorganic biomaterials in 3D printing of scaffold for tissue engineering. Recently, combinations of those polymers and ceramics have been tried to optimize their properties for 3D bioprinting.

Combination of 3D bioprinting and micro/nano-technology has been utilized in tissue engineering area, being considered as a unique technology to overcome the issues of fabrications of biomimetic scaffolds for complex tissues. Nano/micro-technology has been employed for designs of scaffolds as described in following session. Controlling of porosity, permeability, surface properties, initial mechanical properties, and biodegradation of repeating units in polymer’s backbone chains are examples of employment of nano/micro-technology in scaffold designs [24–27]. 3D bioprinting uses hydrogel as a cell carrier and requires properties such as high cell viability, surface tension, water contents and printability with high resolution with tens to hundreds of μm [28]. It provides new approaches towards better and smarter tissue engineering that have the possibility to profoundly impact therapeutic regenerative medicine through better biomimetics of specific tissue structures by nano/micro-technology. Since cells and their components are in the ranges of tens to hundreds micro-meters to nano-meters, cell-biomaterials interactions are affected by those ranges, indicating the importance of biomaterials designs and fabrications through combination of micro/nano-technology and biomimetics. Important factors in biomimetics-based scaffolds designs could be controlled delivery of bioactive molecules, fabrications of right pore size, volume and geometry similar to target ECM as well as biomaterials compositions and physical/chemical properties of the scaffolds [29–32]. Biomimetic scaffolds have been reported having defined mechanical/structural properties of bone tissues such as properties of the cancellous bone’s 50 – 90 volume % porosity and the cortical bone’s dense outer layer of bone with less than 10 volume % [11, 12]. In cartilage, example of combination of biomimetics and nano/micro-technology is designing the structures of cartilage tissues through 3D bioprinting as well as their bio-related chemical and mechanical functions [30]. These functional designs of scaffolds with controlled delivery of biosignals have been expected to lead to better regeneration of various zones of osteochondral ECM.

Even though remarkable progresses was made in tissue engineering by using both 3D bioprinting and nano/micro-technology, there are still huddles, such as the issues of adequate printable biomaterials, biocompatibility, structural stability, biodegradation and immunology, to be overcome in the area of clinical applications to tissue engineering of bone and cartilage among many target tissues. Better cooperation of biomaterials scientists, engineers and clinicians should be made to achieve progress of 3D bioprinting and bone and cartilage tissue engineering.

### Research trends of biomimetic medical materials: micro/nano-patterning technology

There exist largely two approaches to make micro/nano patterns. One is top-down approaches; They use usually the templates which are fabricated through UV photolithography, nanolithography [33–35] such as e-beam lithography, nanoimprint lithography and focused ion beam (FIB) milling, and so on. However, the nanolithography-based top-down approaches for nanoscale patterns suffer from low throughput and high cost. Therefore, a simple and cost-effective process with high throughput is required for the fabrication of well-defined micro/nano patterns. The other approaches, which are called as bottom-up approaches, are generally free from complex template fabrication. They include electrospinning, self-assembly, wrinkle and crack through PDMS stretching, electrodeposition and sol-gel synthesis with anodic aluminum oxide (AAO) or copolymer templates [36–41]. They can make micro/nano patterns in a simple, high-throughput and cost-effective manner, but suffer from obtaining the ordered and reproducible patterns when compared with those of the top-down approaches.

Many researches have been reported about development of micro/nano patterns for biomedical applications using both top-down and bottom-up approaches [42–45]. Specially, polymer-based micro/nano patterns have been widely used for those fields because polymer-based materials have the advantages of low cost, good biocompatibility, high optical clarity and high impact strength [46–48]. Recently, biological materials such as diatom [47] and galium aparine [48] are increasingly used as templates for replica molding process, which enable us to fabricate micro/nano patterns and features with low-cost and high reliability. Also, conventional direct etching process to biomedical materials such as titanium (Ti) is effective to obtain micro/nano structures on its surface [49]. The successful construction of micro/nano-textured surface on a Ti substrate could induce cell adhesion and proliferation efficiently. Recently, nanostructured biomaterials with physical nano-features (nanocrystals, nanofibers, nanocomposites, etc.) have been widely studied in the fields of regenerative medicine due to the resemblance of physical nano-features to natural ECM [50, 51]. Since natural biological tissues, organs and cells have nanoscale features, the biomimetic features and excellent physicochemical properties of nanostructured biomaterials play a key role in stimulating cell growth as well as guiding tissue regeneration [52]. Therefore, the surface designs in nanoscale and molecular level are very important in developing new functional biomaterials.

### Biomimetic medical materials trends: surface modifications

Natural materials like bone, ligaments, wood, shells, and scales are remarkably efficient in terms of fulfilling complex and multiple functional requirements [53]. The development of biomaterials has recently focused on the design of biomimetic materials that are capable of eliciting specific cellular responses and directing new tissue formation mediated by bioactive molecules recognition, which can be manipulated by altering design parameters of the material [54]. For example, an implant associates osteoinductive biomolecules, such as growth factors, hormones, enzymes and DNAs, with osteoconductive calcium phosphates, could regulate and accelerate the bone formation.

Extensive studies have been performed to render materials’ biomimetic. The surface modification of biomaterials with bioactive molecules is a simple way to make biomimetic materials [55]. There are various techniques to immobilize bioactive molecules onto the surfaces of biomaterials, including physical adsorption [56], chemically covalent bonding [57], and biomimetic incorporation [58]. Biomimetic coating has been first introduced by Kokubo in 1990s through soaking alkali treated implants in a simulated body fluid (SBF: ion concentrations similar to human blood plasma) resulting in growth of a bone-like apatite layer [59]. The generated minerals, being biodegradable, porous, and microcrystalline, have the ability to load bioactive molecules onto substrate without compromising their bioactivity. As consequence, the bioactive molecules are truly incorporated into the crystal lattice, not superficially adsorbed upon surfaces. The molecules are released gradually from these coating, rather than in a single rapid burst. One remarkable advantage of the biomimetic coating is that it can even grow on three-dimensional scaffolds.

Surface modification with bioactive molecules offers the potential to control cell behavior only on the surface of biomedical devices. With bulk modification of biomaterials, bioactive molecules are incorporated into the biomaterials and the resulting recognition sites are present not only on the surface but also in the materials [60]. It is well known that the formation of a turban shell includes a process where inorganic minerals deposited on assembled organic proteins from an aqueous solution at mild temperature and pressure [61]. If this type of biomimetic approach could be reproduced, then the bioactive molecules could be incorporated stably in inorganic materials without losing their biological activity [62]. Various bioactive molecules, such as collagen [63], silk fibroin [64], dentin matrix protein 1 [65], chitosan [66], alginate [67], peptide-amphiphile molecular [68], have been served as templates to regulate the growth of inorganic crystals. The structures of the templates play a vital role in controlling the morphologies of the inorganic crystals [69]. For example, fibrous templates lead to a needle-like hydroxyapatite [70], while spherical templates result in sheet-like hydroxyapatite [71]. Simultaneous applying different templates in the system may lead to more sites of nucleation compared with a single-template system, which may affect the orientation, size, and morphology of regulated inorganic crystal.

Biomimetic medical materials with immobilized bioactive molecules possess unique properties of organic and inorganic components in one material for academic research as well as for the development of innovative industrial applications. Thus, the surface modification of metal or bulk modification of ceramic with bioactive molecules is necessary to produce high-performance biomimetic medical materials.

### Recent research trends of articular cartilage repair: cells, scaffolds and cytokines

The predominant approach for tissue regeneration is cell delivery, either by transplantation of progenitor cells or more mature cells. Many cell sources have been explored for the treatment of cartilage defect whether they are either autologous or allogenic. Autologous cells that are clinically available for articular cartilage repair are chondrocytes. Autologous chondrocyte implantation (ACI) was first published in 1994 by Brittberg and it has been expected to show far better results than the marrow stimulating procedure [72, 73]. Autologous chondrocyte implantation, however, was also not perfect and showed the results comparable to or slightly better than those of marrow stimulating procedure in several studies [74]. Fibrous cartilage regeneration, difficulty in procedure and high cost for cell preparation are hurdles in widespread use of this technique [75]. More cell sources including autologous bone marrow cells and adipose stem cells are under investigation and clinical trials, although the evidences are limited so far [76]. Cartilage itself has been shown to have stem cell fraction in their matrix, but they are too few and little has been known about their biological potentials in regenerating tissue [77].

Allogenic cells can be used after ex vivo culture and considered to be different from autologous cells in their behaviors in vivo [78]. These allogenic cells remain as an attractive alternative that circumvents the problem of donor cell shortage. There are, however, technical problems such as immunogenicity, pathogen transmission and mismatch of donor-host micro-environment, which should be resolved prior to clinical applications. Recently allogenic cord blood stem cells have been commercialized in Korea to repair articular cartilage repair for chondral defects as well as degenerative articular diseases. Although the efficacy compared with other regenerative techniques is still in questions, its safety and the minimal efficacy have been accepted by the government agency for permission to be used for patients with chondral defects.

In contrast to the exogenously administered/injected cells, a micro-fracture technique has been considered as a representative surgical procedure that uses endogenous cells in cartilage repair [79]. Simple micro-fractures or subchondral drilling provides a complete set of constituents required for tissue-engineered cartilage regeneration theoretically. However, incomplete healing of the defects, deterioration of the fibrocartilage, and subchondral bone hypertrophy after micro-fracture resulted in unsatisfactory clinical outcomes that necessitated improvement of the methods or the development of novel sources of constituents [80, 81]. Various biomaterials scaffolds that provide temporary compartment to retain cells and enable diffusion of vital nutrients were developed to enhance potentials of tissue regeneration by micro-fracture techniques [82]. Autologous bone marrow cells can be harvested from ilium, concentrated and then applied, thus providing higher number of cells to participating tissue regeneration [83]. Their results are controversial and well-designed level 1 studies are scarce, but several promising results showed potentials of their applications. It is still controversial whether the cultured chondrocytes are better than endogenous bone marrow cells. Several randomized clinical trials showed no differences in outcomes between micro-fracture and ACI, while others did although it is small [84].

Polymeric biomaterials scaffolds were used to eliminate complications and to promote the outcomes of ACI [85]. Collagen sheets and hyaluronic acid gels among other scaffolds were expected to reduce the hypertrophic complications of periosteum that was used for ACI. In general, the 2^nd^ and 3^rd^ generation ACIs that used biomaterial scaffolds showed tendency toward better outcome than its 1^st^ generation did. Scaffolds such as hyaluronan have been used to enhance the outcomes of micro-fracture. Autologous matrix-induced chondrogenesis (AMIC) showed better mid to long term results than micro-fracture only did [82].

Collagen and fibrin also function as bioactive cues like growth factors or cell recruitment factors. Bioactive cues to initiate and enhance the regeneration potential have been considered as one of the most important issues that should be addressed. Whereas the cell transplantation is still the predominant approach, emerging studies have shown enhanced potentials of host endogenous cells. Cell homing is the concept that cells such as mesenchymal stem cells (MSCs) migrate to injured tissue as MSCs express various chemokine receptors and adhesion molecules [86]. Its attracting factors are numerous. Recently an acellular ‘smart’ scaffold with TGF-β_3_ has been reported to regenerate whole synovial joint including bone, cartilage and synovium [87]. Also, poly(lactide-*co*-glycolide) (PLGA) scaffolds seeded with autologous endothelial progenitor cells have shown to repair a full-thickness osteochondral defect in rabbits [88].

Cell transplantation and cell homing are not exclusive each other in cartilage tissue engineering. Furthermore, engineered scaffolds are required to provide a provisional space for effective tissue regeneration as well as manipulation of appropriate bio-environments.

## Conclusions

Four topics of biomimetic medical materials from different research groups with different backgrounds have been discussed such as 1) 3D bioprinting focusing on medical materials, 2) designs of nano/micro-technology, 3) surface modification of biomaterials for their interactions with cells, and 4) clinical aspects of biomaterials for cartilage focusing on cells, scaffolds and cytokines. Even though traditional methods of fabrications of tissue engineering scaffolds have still many advantages, 3D printing methods and right biomaterials have been emerged as extremely important factors in tissue engineering, where both designs of biomaterials in micro/nano levels and surface modifications have acted as the basic technologies in designs of biomimetic materials. Combination of 3D printing and stem cells are extremely important to tissue engineering of bone and cartilage tissues.
